# Efficiency of fluorescent cholangiography during laparoscopic cholecystectomy for subvesical bile ducts: A case report

**DOI:** 10.1016/j.ijscr.2019.03.042

**Published:** 2019-03-30

**Authors:** Hirotaka Kitamura, Toshikatsu Tsuji, Daisuke Yamamoto, Tohru Takahashi, Shinichi Kadoya, Masaru Kurokawa, Hiroyuki Bando

**Affiliations:** Department of Gastroenterological Surgery, Ishikawa Prefectural Central Hospital, 2-1 Kuratsuki-Higashi, Kanazawa, 920-8530, Japan

**Keywords:** FC, fluorescent cholangiography, LC, laparoscopic cholecystectomy, DIC-CT, drip-infusion cholangiography with computed tomography, ICG, indocyanine green, CT, computed tomography, IGFI, indocyanine green fluorescence imaging, IOC, intraoperative cholangiography, Duct of Luschka, Hepaticocholecystic duct, Aberrant bile duct, Bile duct injury, Fluorescence imaging

## Abstract

**Introduction:**

The subvesical bile ducts are located in the peri-hepatic connective tissue of the gallbladder fossa. Injury of the subvesical bile ducts provokes the severe complication of bile leak. Until now, fluorescent cholangiography has been employed during hepatobiliary surgery. Herein, we report the detection of subvesical bile ducts by fluorescent cholangiography during laparoscopic cholecystectomy.

**Presentation of case:**

A 63-year-old female was admitted to our department for surgery for symptomatic cholelithiasis. The subvesical bile ducts were not observed on drip-infusion cholangiography with computed tomography. Immediately following induction of anesthesia, 2.5 mg of indocyanine green was intravenously injected. Fluorescent cholangiography demonstrated two thin aberrant bile ducts during dissection of Calot’s triangle. We considered them to be subvesical bile ducts. We ligated them with clips, divided them, and then performed laparoscopic cholecystectomy using a standard procedure. The patient had a good post-operative recovery without bile leakage. Postoperative laboratory test results were all within normal limits. Computed tomography revealed no dilatation of the intrahepatic bile duct after laparoscopic cholecystectomy. The patient was discharged on postoperative day 4.

**Discussion:**

Injury to the subvesical bile ducts is one of the most common causes of bile leakage associated with cholecystectomy. Fluorescent cholangiography enabled real-time identification of the thin subvesical bile ducts, which were undetectable by drip-infusion cholangiography with computed tomography.

**Conclusion:**

Fluorescent cholangiography during laparoscopic cholecystectomy may be useful for preventing postoperative bile leakage.

## Highlights

• The subvesical bile ducts are important from the potential risk for bile leakage.
• It is difficult to identify the subvesical bile ducts intraoperatively.
• Fluorescent cholangiography visualized the subvesical bile ducts clearly.

## 1 Introduction

Accurate knowledge of the anatomy of the bile duct, which is well-known to have structural variation, is important for surgeons performing cholecystectomy. One common variation in the bile duct is the subvesical bile ducts, but there is no consensus on its detailed anatomy. Injury to the subvesical bile ducts is one of the most common causes of bile leakage associated with cholecystectomy [1]. Currently, fluorescent cholangiography (FC) after intravenous injection of indocyanine green (ICG) is a promising new technique for improved intraoperative recognition of biliary anatomy [2]. FC is non-invasive as the biliary structures are not punctured and X-ray radiation is not required [3,4]. The case presented here describes the detection of the subvesical bile ducts by FC during laparoscopic cholecystectomy (LC).

This manuscript has been reported in line with the SCARE guidelines [5].

## 2 Case presentation

**Fig. 1:**
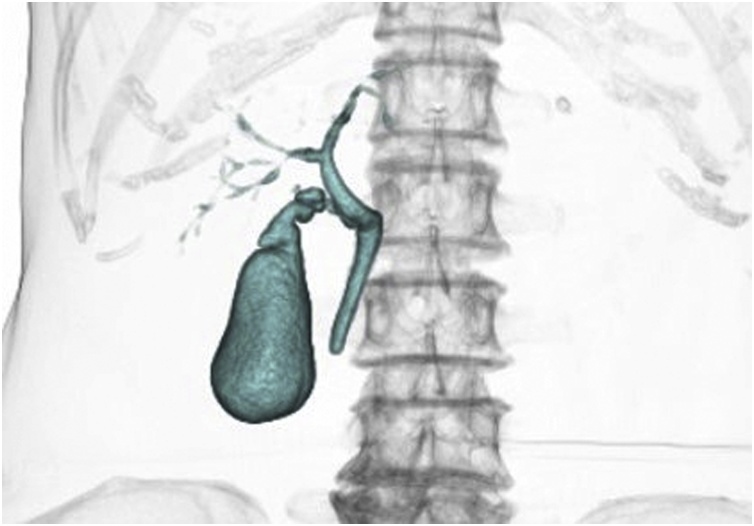
Drip-infusion cholangiography with computed tomography findings. The subvesical bile ducts were not visualized clearly by preoperative drip-infusion cholangiography with computed tomography.

A 63-year-old female presented to our hospital with right hypochondrium pain. She underwent appendectomy at the age of 20 years and had an uneventful recovery following that. Laboratory results showed AST of 34 U/L; ALT of 32 U/L; and ɣ-GTP of 142 U/L. A gallstone was found on computed tomography (CT) and abdominal ultrasonography. By drip-infusion cholangiography with computed tomography (DIC-CT) performed before LC, we were able to clearly visualize the cystic duct, common hepatic duct, right anterior sectional duct, and right posterior sectional duct, but the subvesical bile ducts were not visualized (Fig. 1). She was scheduled to undergo LC for symptomatic cholelithiasis.

**Fig. 2:**
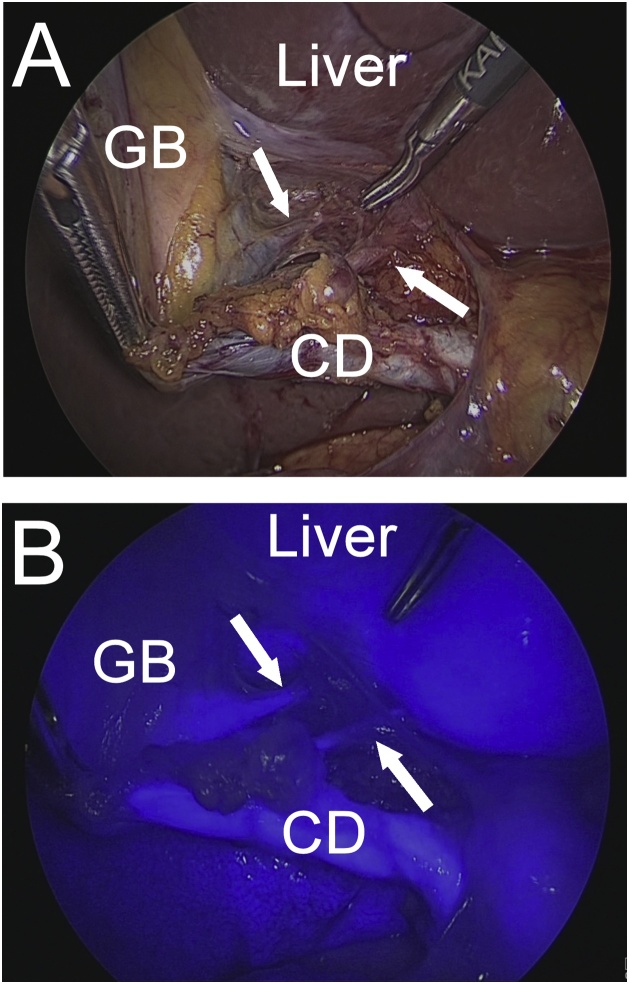
The laparoscopic view of Calot’s triangle. (A) Laparoscopic view of the subvesical bile ducts under normal light. (B) The subvesical bile ducts were clear on fluorescent cholangiography. CD; cystic duct, GB; gallbladder, Arrow; subvesical bile ducts.

One milliliter (2.5 mg/ml) of ICG was injected intravenously prior to beginning the surgery. The D-light P system (KARL STORZ, Germany) with the integrated indocyanine green fluorescence imaging (IGFI) mode was prepared to visualize the bile duct. The operative field was inspected in the IGFI mode before dissection of Calot’s triangle. The common bile duct and cystic duct were visualized on FC. LC was performed using a standard procedure under normal lighting. During dissection, FC was used when needed until the critical view of safety was confirmed. FC detected two aberrant bile ducts, 1 to 2 mm in diameter, during the dissection of Calot’s triangle (Fig. 2). We achieved the critical view of safety, and considered these ducts to be the subvesical bile ducts. After division of the cystic duct and cystic artery, we ligated the subvesical bile ducts with clips and divided them. Then, FC was used again to evaluate bile leakage. Dissection of the gallbladder from the liver bed was continued and the gallbladder was resected.

**Fig. 3:**
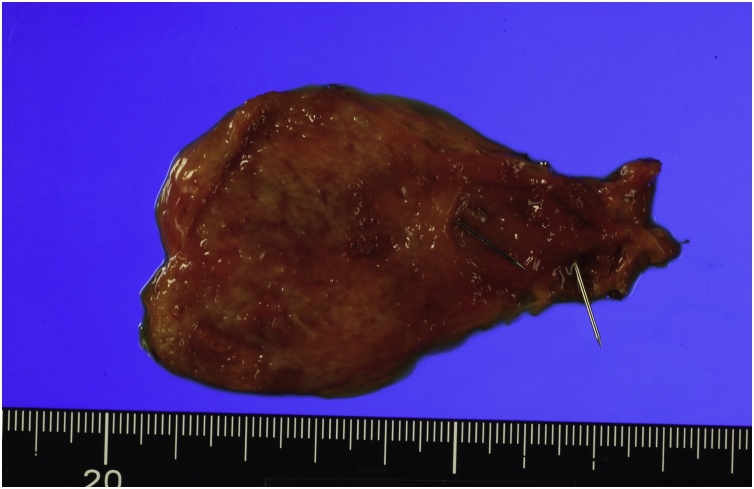
The resected specimen. Nails were inserted in the subvesical bile ducts of the resected specimen.

The resected specimen revealed that two subvesical bile ducts drained into the gallbladder (Fig. 3). Postoperative laboratory test results were all within normal limits. CT demonstrated no dilatation of the intrahepatic bile duct after LC. The patient was discharged uneventfully on the fourth postoperative day.

## 3 Discussion

The subvesical bile ducts are located in the peri-hepatic connective tissue of the gallbladder fossa. There is no consensus regarding the anatomy of these aberrant bile ducts. They are considered by some to be small bile ducts that drain directly into the body of the gallbladder, whereas others consider them to be networks of miniscule bile ducts between the liver capsule and the gallbladder [1]. Although the anatomical details of these ducts remain unclear, their prevalence has been reported to be 4% [1].　In our case, we considered the subvesical bile ducts to have drained directly into the gallbladder, but we were unsure of their origin because they were not visible on DIC-CT and we did not perform intraoperative cholangiography (IOC).

The subvesical bile ducts are important from a clinical perspective and poses a potential risk for injury during cholecystectomy [1,6,7]. Recent studies have suggested that postoperative bile leakage requiring treatment occurs in approximately 0.72% to 0.9% of patients [8,9]. The frequency of clinically relevant bile leakage caused by inadvertent injury to the subvesical bile duct was reported to be approximately 27% [1].

FC has been suggested as an alternative technique for improved intraoperative recognition of the biliary anatomy. Osayi et al. reported that after complete dissection, the rates of visualization of the cystic duct, common bile duct, and common hepatic duct using FC were 95.1%, 76.8%, and 69.5%, respectively, compared with 72.0%, 75.6%, and 74.3%, respectively, by IOC [4]. Boni et al. were able to identify the biliary anatomy in all 52 cases using FC, including the cystic duct-common bile duct junction [10]. Although IOC is the most common technique for intraoperative visualization of the extrahepatic bile duct, its routine use for prevention of bile duct injury is controversial [8]. However, IOC allows for early identification of bile duct injury as long as it is correctly interpreted [8]. On the other hand, IOC cannot depict all details such as subvesical bile ducts leakage [6]. Precise knowledge of anatomical variations in the biliary tree and careful pre-operative evaluation are key to safe and satisfactory LC. Pre-operative magnetic resonance cholangiopancreatography (MRCP) and DIC-CT can precisely depict the biliary tree. Kitami et al. identified 32 subvesical bile ducts in 28 (10.1%) of 277 patients by DIC-CT [11]. In our case, the subvesical bile ducts were visualized by FC during LC, but were unclear by DIC-CT.

## 4 Conclusion

FC during LC may be useful for preventing leakage from the subvesical bile ducts.

## Conflicts of interest

Dr. Hirotaka Kitamura, Dr. Toshikatsu Tsuji, Dr. Daisuke Yamamoto, Dr Tohru Takahashi, Dr. Shinichi Kadoya, Dr. Masaru Kurokawa, and Dr. Hiroyuki Bando have no conflict of interest of financial to disclose.

## Funding

This research did not receive any specific grant from funding agencies in the public, commercial, or not-for-profit sections.

## Ethical approval

This study design was approved by the Ishikawa Prefectural Central Hospital Ethics Committee (approval no. 1202).

## Consent

Written informed consent was obtained from the patient for publication of this case report and accompanying images. A copy of the written consent is available for review by the Editor-in-Chief on this journal on request.

## Author contribution

Study concept and design: HK, MK, HB.

Writing original draft, review and editing: HK.

Final approval of the version to be published: HK, TT, DY, TT, SK, MK, HB.

## Registration of research studies

This case report was a part of single-arm study which was approved by the ethics committee of our hospital, and written informed consent was obtained from the patient. The protocol was registered with UMIN-CTR under the ID number UMIN000027725.

## Guarantor

Hirotaka Kitamura, corresponding author of this article.

## Provenance and peer review

Not commissioned, externally peer-reviewed.
